# Neutral buoyancy is optimal to minimize the cost of transport in horizontally swimming seals

**DOI:** 10.1038/srep02205

**Published:** 2013-07-16

**Authors:** Katsufumi Sato, Kagari Aoki, Yuuki Y. Watanabe, Patrick J. O. Miller

**Affiliations:** 1 International Coastal Research Center, Atmosphere and Ocean Research Institute, The University of Tokyo, 5-1-5 Kashiwanoha, Kashiwa City, Chiba Prefecture, 277-8564, Japan; 2 National Institute of Polar Research, Tachikawa, Tokyo 190-8518, Japan; 3 Sea Mammal Research Unit, School of Biology, University of Saint Andrews, Fife, KY16 8LB, Scotland

## Abstract

Flying and terrestrial animals should spend energy to move while supporting their weight against gravity. On the other hand, supported by buoyancy, aquatic animals can minimize the energy cost for supporting their body weight and neutral buoyancy has been considered advantageous for aquatic animals. However, some studies suggested that aquatic animals might use non-neutral buoyancy for gliding and thereby save energy cost for locomotion. We manipulated the body density of seals using detachable weights and floats, and compared stroke efforts of horizontally swimming seals under natural conditions using animal-borne recorders. The results indicated that seals had smaller stroke efforts to swim a given speed when they were closer to neutral buoyancy. We conclude that neutral buoyancy is likely the best body density to minimize the cost of transport in horizontal swimming by seals.

Efficient locomotion between two distant points (e.g. breeding and foraging sites) is important for migrating animals. A recent paper indicated that negatively buoyant aquatic animals (sharks and pinnipeds) sometimes adopted vertical undulations with alternating glide and powered locomotion, and concluded the undulating movements reduced mechanical cost compared with continuous swimming^1^. At first glance, the reported gait pattern resembles the undulating flights of birds, which can reduce mechanical cost of horizontal flight^2^. However, two different mechanisms of gliding observed in the aquatic animals were not distinguished in the paper^1^.

In aquatic divers, both stroke-and-glide swimming and prolonged gliding followed by active swimming^3^,^4^,^5^,^6^,^7^,^8^,^9^ incorporate periods of passive movement. However, in the latter case, net buoyancy is equilibrated with hydrodynamic drag, and swimming speed thereby converges to a terminal speed (potential energy is consumed for work against drag) during the gliding phase of the dive. In contrast, the stroke-and-glide gait is governed by different physical mechanisms: the animal gains kinetic energy by thrusting and loses kinetic energy when gliding that does not require any change in potential energy. Thus swim speed fluctuates periodically. Depending upon the body angle of motion during stroke-and-glide swimming, both kinetic and potential energy can change throughout a glide. In the right conditions, short periodical intermittent stroking (stroke-and-glide) can contribute to energy saving in flying and swimming animals^2^,^10^,^11^.

A theoretical study first suggested a possibility of energy saving in negative buoyant fish by prolonged gliding with a gradual increase of depth then actively swimming upwards to the original depth^12^. Gleiss et al. (2011) discussed that potential energy from gravity and altitude is translated into horizontal distance via gliding, which is thought to result in energy saving compared with continuous level transit^1^. For prolonged gliding, this is true in the descent direction aided by negative buoyancy, but costs are naturally greater in the opposite direction^13^. Whether or not such patterns of long-duration gliding and subsequent active upward swimming yield mechanical savings for horizontal transit is not clear^14^.

Gleiss et al. (2011) found reduced swimming efforts of an elephant seal during undulating locomotion at the bottom phase of u-shape dives (in Fig. 3e in their paper), and attributed the savings to greater assumed negative buoyancy^1^. However, comparison of swimming efforts between conditions known to differ in body density are needed to test that conclusion. We used data obtained from two field experiments with three Northern elephant seals *Mirounga angustirostris* and one Baikal seal *Phoca sibirica* during which body density was manipulated by detachable weights and floats^8^,^15^. For each seal, we were able to precisely replicate their analysis^1^ with a high degree of certainty about the body density of the seal in different conditions. In the present study we evaluate the effect of body density on stroke efforts of seals and consider what might be the optimal body density to minimize the cost of transport during horizontal swimming.

## Results

**Figure 1:**
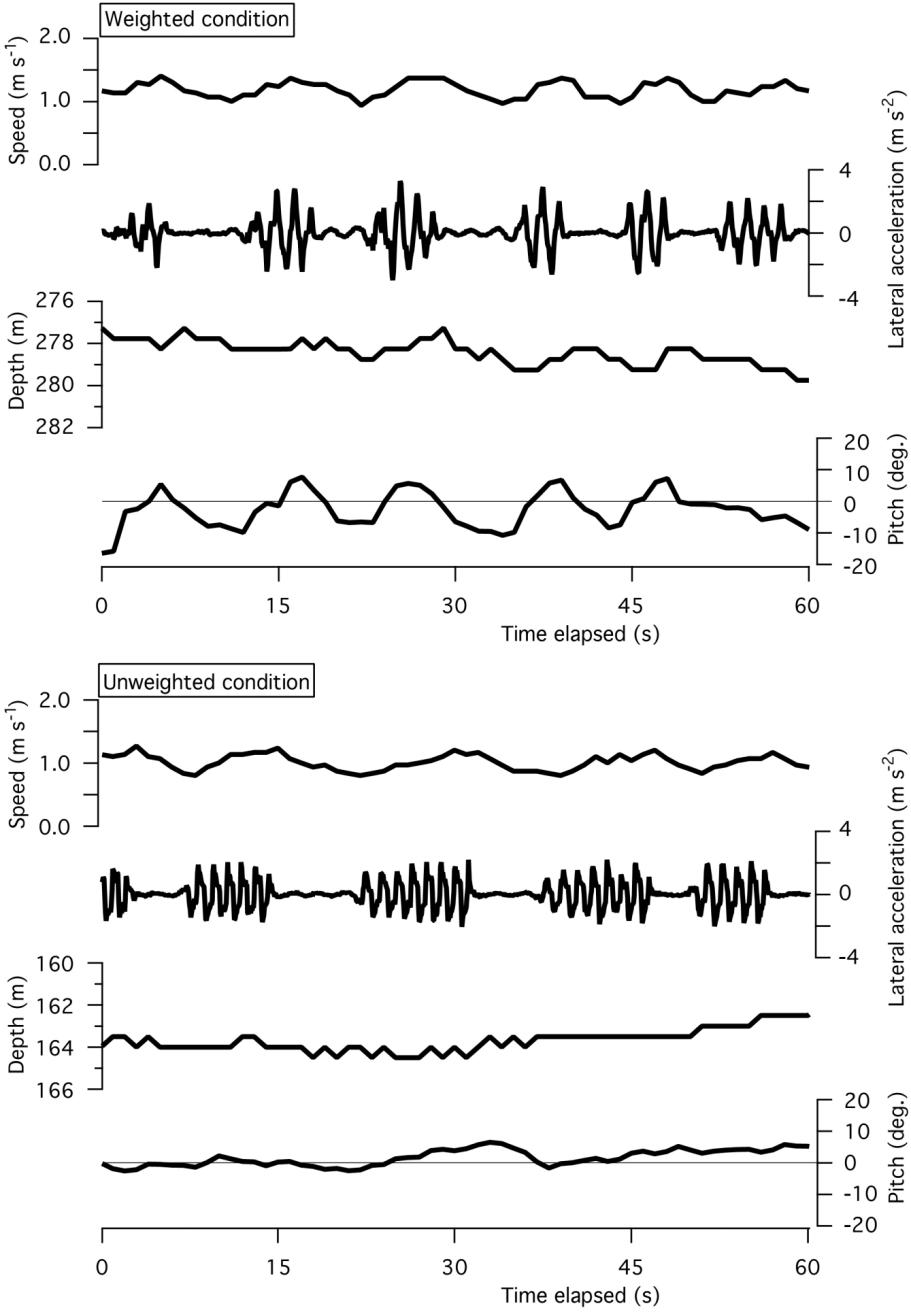
Two examples of intermittent strokes by a Northern elephant seal (no. 4 in Table 1) in weighted and unweighted conditions. Data was recorded in a field experiment conducted near Ano Nuevo, California, USA^15^.

**Figure 2:**
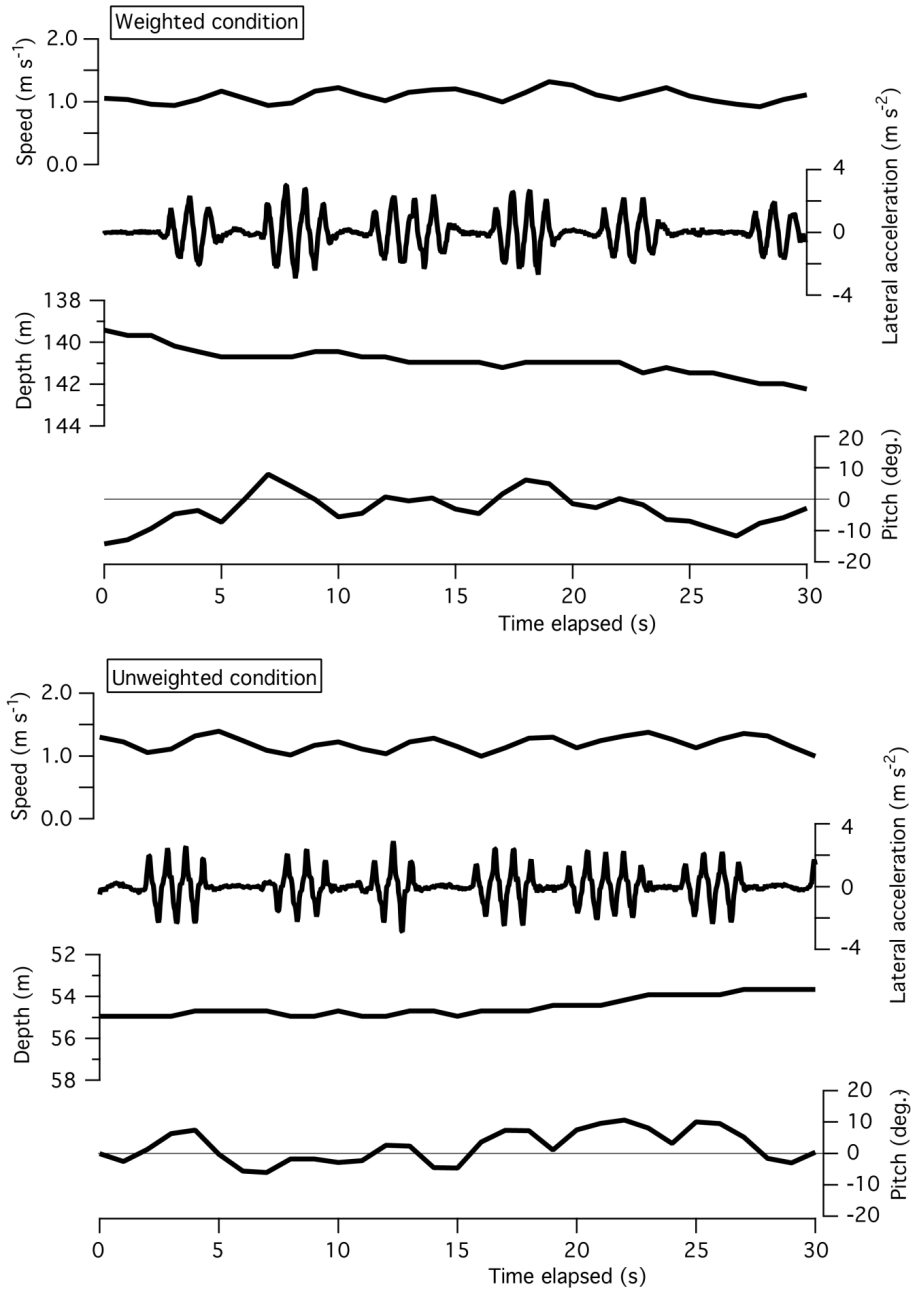
Two examples of intermittent strokes by a Baikal seal in weighted and unweighted conditions. Data was recorded in a field experiment conducted at Lake Baikal, Russia^8^.

Figure 1 indicates examples of intermittent strokes by a Northern elephant seal (no. 4 in Table 1) swimming within a narrow range of depth with and without an attached weight. Swim speed fluctuated with the stroke-and-glide pattern, but undulation in depth was not apparent. Pitch angle seemed to undulate when the seal was weighted (estimated body density was 29.8 kg m^−3^ larger than seawater density). Pitch became positive when the seal stroked and negative when it glided (Fig. 1 upper panel). However, this undulation in pitch angle was not apparent after the weight was detached (body density deviation from the seawater decreased to 12.1 kg m^−3^). A similar tendency was observed in a Baikal seal (Fig. 2). The stroke-and-glide swimming was accompanied with undulation in speed, not in depth. The undulation in pitch angle can be seen in weighted and unweighted conditions, in which body density deviations from fresh water were 43 and 15 kg m^−3^, respectively.

**Figure 3:**
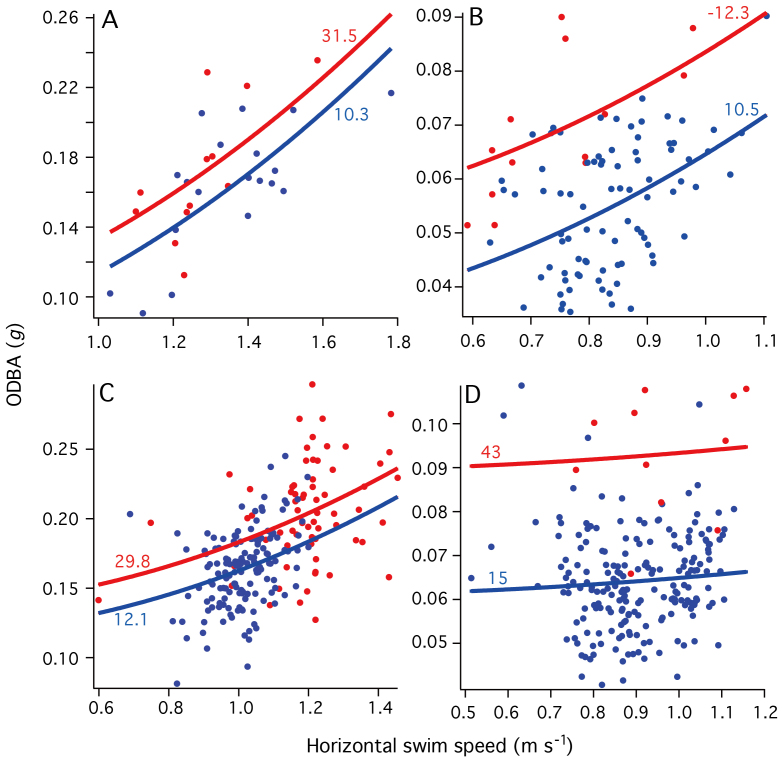
Stroke efforts in relation to horizontal swim speed of three Northern elephant seals ((A): elephant seal 1, (B): seal 2, (C): seal 4) and one Baikal seal (D). The effort was represented by overall dynamic body acceleration (ODBA)^1^. Color of plot indicates density conditions (red: more deviated from neutral buoyancy, blue: closer to neutral buoyancy). Curves were derived from the statistical models calculated for each density condition. Values by the curves are density deviation from water density (see Table 1).

A total of 554 stroke-and-glide cycles of mean duration ranging from 4.9 to 14.9 s were identified for analysis of stroking efforts (Table 1). Mean horizontal swim speeds of the three elephant seals and one Baikal seal ranged from 0.8 to 1.3 m s^−1^. Stroke efforts of seals increased with swim speed (Fig. 3) and a statistical model including both squared horizontal swim speed and absolute density deviation from neutral buoyancy was selected with minimum AIC (Table 2). Seals of smaller density deviation (blue plots in Fig. 3) had lower efforts in comparison with seals of larger density deviation (red plots in Fig. 3). For a Baikal seal, a statistical model including only absolute density deviation was also selected with minimum AIC (Table 2). However, the interpretation was the same: the Baikal seal had smaller stroke efforts when it was unweighted and the body density deviated less from neutral buoyancy. These results indicate that seals had smaller stroke efforts to swim horizontally at a given speed when their body densities were closer to neutral buoyancy.

## Discussion

For flying birds, vertical undulation during periods of stroke-and-glide flying is common because of upward lift produced by wing flapping and downward gravity during glides, particularly during glide phases of ‘bounding flight’ during which they do not extend their wings, apparently to minimize drag^2^. However, in cases of aquatic animals such as seals used in the present study, weight in the water is mostly supported by buoyancy, as a result, vertical undulation during periods of stroke-and-glide swimming was not apparent in comparison with flying animals. Gleiss et al. (2011) indicated examples of intermittent strokes that were accompanied with vertical undulation observed in a southern elephant seal (Fig. 3bd in their paper)^1^, however, our data indicated that periodical variation in depth was less than or equal to the 0.5 m resolution of the depth sensor in stroke-and-glide swimming by seals though periodical variation in speed was common (Figs.1 and 2) as for undulating flight by birds^2^. In our data, stroke-and-glide gait was observed not only in weighted (or floated) conditions (more different from neutral buoyancy) but also in unweighted (or floated-and-weighted) conditions (closer to neutral buoyancy), which means that non-neutral buoyancy was not essential for intermittent stroking.

Our results showed that horizontal swimming effort of seals was consistently higher in conditions when body density deviated more from neutral buoyancy (Table 2 and Fig. 3). We could recognize some undulations in pitch angles of non-neutral buoyant seals during stroke-and-glide swimming (Figs. 1 and 2). These undulations may have increased the cost of horizontal swimming. We conclude that non-neutral buoyancy is neither essential nor beneficial for intermittent stroking, and that stroke-and-glide horizontal swimming should be most efficient when the animal is close to neutral buoyancy.

## Methods

### Field experiments

In the present study, we used data recorded in two field experiments, in which free-diving Northern elephant seals and a Baikal seal were instrumented in California, USA (2008), and in Lake Baikal, Russia (2005), respectively. Experiments were performed in accordance with relevant guidelines and regulations in each study site. Details of the experimental procedure were described in published papers^8^,^15^ and the procedures were approved by the UCSC CARC (IACUC) committee and permitted under NMFS marine mammal permits nos 786–1463 and 87–143. In both experiments, a multi-sensor data logger (3MPD3GT; 26 mm diameter, 175 mm length, 140 g in air; Little Leonardo Co., Tokyo, Japan) was deployed on seals to record depth and swim speed at 1 s intervals, and three-axis accelerations for detecting pitch and hind flipper movements at 1/16 (elephant seal) or 1/32 s (Baikal seal) intervals.

The body densities of elephant seals were experimentally changed by attaching a weight and float just behind the data logger on the back of seals. A time-scheduled release mechanism (Little Leonardo Co.) was used to automatically release the weight and float. The front part of the weight and the float were covered with a streamlined plastic housing to provide a consistent alteration in frontal area for each seal without changing the drag coefficient between conditions^15^. We obtained three buoyancy manipulation conditions: floated-and-weighted, weighted and floated (Table 1). In case of the Baikal seal, weight was deployed on the seal and was detached 24 h after deployment by the release mechanism.

### Data analysis

The body composition of each elephant seal was estimated by isotope dilution, and then the body density was calculated from proportion and the density of each component (lipid, protein, ash and body water)^15^. The body density of the Baikal seal was estimated from deceleration of swim speed and pitch angle^8^. Absolute differences between body and water densities (Table 1) were used for statistical analysis.

Swim speed was recorded as the number of rotations per second (rev s^−1^) of an external propeller mounted at the anterior end of the multi-sensor data logger. The rotation value was converted to actual swim speed (m s^−1^) using the calibration method^5^. Effort of seals were represented by overall dynamic acceleration ODBA, which is an index to represent acceleration due to motion of the body, calculated from 3-axis acceleration data as described in the preceding paper^1^.

We extracted individual undulations when a stroke-and-glide cycle began and ended at the same depth^1^. Since the majority of stroke-and-glide cycles were not accompanied with vertical undulations, stroke-and-glide cycles were also extracted during horizontal swims when depth change was equal to or less than 0.5 m (resolution of depth sensor). Then, we calculated the mean ODBA and mean swim speed during those cycles. We adjusted the mean swim speed to horizontal speed using the absolute pitch angle calculated from longitudinal acceleration.

### Statistical analysis

To determine an effect of body density on stroke effort, linear models were fitted to the data of each seals, in which ODBA was a response variable, and squared horizontal swim speed and absolute density deviation were fixed factors. Squared speed was fit in the models to account for the non-linear increase in resistive drag forces due to speed. Akaike Information Criteria (AIC) were compared between the fitted models to select the most parsimonious model. For the model fitting, R 2.6.2 (R Foundation for Statistical Computing, Vienna, Austria) was used with the *glm* function in R package.

## Author Contributions

K.S. and P.J.O.M. led the study. Y.Y.W. and P.J.O.M. designed and conducted field studies. K.A. analyzed data. K.S. and P.J.O.M. wrote the manuscript; all authors discussed results, and read, edited the manuscript.

## Figures and Tables

**Table 1:** Body conditions of seals and characteristics of all stroke-and-glide cycles (total n = 554)

		Body density	Difference from water density^1^	Stroke-and-glide cycles	
Seal	Condition	(kg m^−3^)	(kg m^−3^)	n	Duration (s)
elephant seal 1	Weighted	1060.1	31.5	12	11.8 ± 4.1
	Unweighted	1038.9	10.3	19	14.9 ± 4.8
elephant seal 2	Floated-and-weighted	1038.2	10.5	85	9.0 ± 2.0
	Floated	1015.4	−12.3	13	12.0 ± 6.8
elephant seal 4	Weighted	1057.9	29.8	66	12.3 ± 3.1
	Unweighted	1040.2	12.1	148	14.0 ± 2.6
Baikal seal	Weighted	1043	43	11	5.5 ± 1.0
	Unweighted	1015	15	200	4.9 ± 0.9

1To calculate density difference, sea water density (1027.7–1028.6 kg m^−3^, estimated from salinity, depth and temperature^15^) was used for elephant seals and fresh water density (1000 kg m^−3^) was used for a Baikal seal. Duration of stroke-and-glide is represented as mean ± s.d.

**Table 2:** AIC value for each linear model describing the effect of body density deviation on stroke efforts. Underlines indicate the minimum AIC of each individual

	Elephant seal			
Model	1	2	4	Baikal seal
Null	−114	−572	−815	−1230
Squared horizontal speed	−128	−579	−895	−1232
Absolute density deviation	−112	−585	−880	−1287
Squared horizontal speed & Absolute density deviation	−130	−606	−906	−1287

Note that the elephant seal 1 had a delta AIC of 2, compared between models including and not including absolute density deviation. It suggests less evidence to select the more complex model, which might be caused by relatively small sampling number (n = 12 + 19) in comparison with other seals (Table 1).
